# How LMX Differentiation Attenuates the Influence of Ethical Leadership on Workplace Deviance: The Mediating Role of Psychological Empowerment

**DOI:** 10.3389/fpsyg.2021.693557

**Published:** 2021-09-30

**Authors:** Yirong Guo, Limei Chen, Lynda Jiwen Song, Xiaoming Zheng

**Affiliations:** ^1^Institute of Education, Xiamen University, Xiamen, China; ^2^Faculty of Business and Economics, University of Hong Kong, Hong Kong, China; ^3^Leeds University Business School, University of Leeds, Leeds, United Kingdom; ^4^School of Economics and Management, Tsinghua University, Beijing, China

**Keywords:** ethical leadership, psychological empowerment, LMX differentiation, deviant behavior, social learning theory

## Abstract

The previous research has mostly proposed that ethical leadership contributed to less deviant behavior; however, recent studies found that this relationship might not always be significant. Therefore, a deeper and more nuanced investigation of how and when ethical leadership influences deviant behavior is highly warranted. In the present research, drawing on social learning theory as our overarching theoretical framework, we posited that high level of LMX differentiation will impede the effect of ethical leadership on employee deviant behavior, and thus, ethical leadership could reduce employees’ deviant behavior in teams with lower LMX differentiation rather than high LMX differentiation. Furthermore, we proposed that the interactive effect of ethical leadership and LMX differentiation on employee deviant behavior is mediated by employee psychological empowerment. More specifically, ethical leadership is more likely to enhance employee psychological empowerment in teams with low LMX differentiation than in teams with high LMX differentiation, and enhanced psychological empowerment contributed to less deviant behavior. Through a multi-source field study *via* 379 paired samples from the southwest of China, we found support for all of our hypotheses. The results’ contribution to research on organizational behavior, limitations in the study, and future directions for researchers are also discussed.

## Introduction

Employee deviant behavior, or workplace deviance, refers to employees’ voluntary actions that violate organizational norms and may potentially cause harm to individuals and/or the property of an organization (Robinson and Bennett, 1995; Robinson and O'Leary-Kelly, 1998), such as deliberately damaging property of organizations, working slow, and saying rude things about others (Robinson and O'Leary-Kelly, 1998). These behaviors have been demonstrated to cause organizations great productivity and property loss, occupy targeted employee serious mental or emotional distress, and even spill over some negative effect to the broader society (Bennett and Robinson, 2000; Ferris et al., 2009; Bennett et al., 2019; Dhanani and LaPalme, 2019). To reduce these behaviors, researchers and managers have strived for years to figure out possible solutions (Dineen et al., 2006; Mackey et al., 2019).

Due to its unique characteristics of being *Moral Persons* and *Moral Managers*, ethical leadership has been recognized as an effective remedy to the problem of workplace deviance (Mayer et al., 2009; Van Gils et al., 2015; Mo and Shi, 2017). Most of the extant research has elucidated such effect from the perspective of social learning (e.g., Mayer et al., 2009; Resick et al., 2013), generally arguing that employees tend to take ethical leaders as role models and learn appropriate behavior from them, such as refraining themselves from harmful behavior to the organization or other employees. However, recent studies found that ethical leadership was not always significantly related to less workplace deviance (e.g., Gok et al., 2017; Babalola et al., 2019), suggesting that the effect of ethical leadership on deviant behavior is more complicated than generally assumed. Therefore, a deeper and more nuanced investigation of how and when ethical leadership influences deviant behavior is highly warranted.

Indeed, according to social learning theory, mere exposure to potential models (e.g., leaders) does not necessarily ensure the acquisition and maintenance of imitative behavior. Instead, four sub-processes (i.e., attention, retention, motor reproduction, and motivation) matter for successful observational learning, as these processes will affect how individuals observe, learn, and develop their own judgments regarding the extent to which they view their leaders as credible, attractive, and legitimate models and then behave as their leaders (Bandura, 1972, 1977). Being a part of the team, employees do not only focus on how they are treated by the team leader, but are also aware of how other members are treated (Lind et al., 1998), based on which to view, interpret, and respond to ethical leaders. Accordingly, this study proposes that leader–member exchange differentiation (LMX differentiation), reflecting the extent of how differentially leaders interact with all team members (Liden et al., 2006), could affect how the above four sub-processes unfold, and thus influence the extent to which employees model after and emulate ethical leaders (Lind et al., 1998; Lind, 2001). To be specific, we postulate that high level of LMX differentiation will impede employees’ attention and retention of ethical leaders’ behavior and demotivate them to learn from leaders, therefore weakening the effect of ethical leadership on employee deviant behavior.

Furthermore, drawing upon social learning theory, we propose that the interactive effect of ethical leadership and LMX differentiation on employee deviant behavior is achieved by employees’ increased psychological empowerment, which manifests as the motivational mechanism underlying the social learning process, including the sense of meaningfulness, self-determination, competence, and impact (Spreitzer, 1995). Specifically, when LMX differentiation is low, employees are more likely to view ethical leaders as credible, attractive, and legitimate models, paying great attention to leaders’ behavior, accurately coding this behavior, and feeling motivated to imitate it, and thus sense more psychological empowerment and finally refrain themselves from deviant behavior.

**Figure 1:**
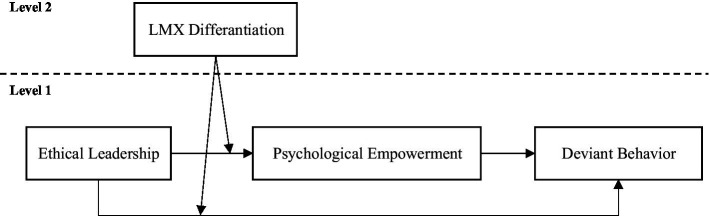
Proposed moderated mediation model linking ethical leadership to deviant behavior.

Hence, this study investigated the relationship between ethical leadership and employee deviant behavior based on social learning theory, proposing that ethical leadership could reduce employees’ deviant behavior in teams with lower LMX differentiation, and this effect is mediated by employee psychological empowerment (see Figure 1). Based on a multi-source and multi-level field study in China, we hope to contribute to the previous research in at least three ways. First, we examine the boundary effect of LMX differentiation on the social learning process wherein employees decrease their deviant behavior through modeling after the ethical leader. In doing so, we challenge the generally held assumption that employees necessarily take ethical leaders as role models and enrich our understanding of the complicated relationship between ethical leadership and deviant behavior. Second, although prior studies have found the psychologically empowering effect of ethical leadership on employees (e.g., Zhu et al., 2004; Zhu, 2008; Dust et al., 2018) as well as the refraining effect of psychological empowerment on employees’ negative behavior (e.g., Kim et al., 2016; Lorinkova and Perry, 2017), extant research exploring the mediating effect of psychological empowerment between ethical leadership and deviant behavior is relatively scarce. Thus, we extend prior research by indicating that psychological empowerment serves as an important and integrative motivational mechanism relating ethical leadership with employees’ deviant behavior. Third, this study explores the effect of ethical leadership on employees’ deviant behavior from the perspective of social learning, answering calls of the previous study to identify how and under what conditions employees learn from ethical leaders (Moberg, 2000).

## Theoretical Framework And Hypotheses

According to the currently accepted definition, ethical leadership means “the demonstration of normatively appropriate conduct through personal actions and interpersonal relationships, and the promotion of such conduct to followers through two-way communication, reinforcement, and decision-making” (Brown et al., 2005). Two essential characteristics constitute ethical leadership: On the one hand, ethical leaders are *Moral Persons*, characterized as being honest and trustworthy, caring about employees, practicing moral standards, and making decisions complied with values and ethical principles; on the other hand, ethical leaders are *Moral Managers* for they communicate with employees, set values and moral standards, and also use rewards or punishments to maintain employees’ moral behavior (Trevino et al., 2000; Brown and Trevino, 2006). Due to these two unique characteristics, previous studies have generally suggested that ethical leadership contributed to decreasing workplace deviance, most of which elaborated this effect from a social learning perspective (e.g., Mayer et al., 2009; Resick et al., 2013). To be specific, drawing upon social learning theory, extant research has concluded that employees generally viewed ethical leaders as role models and learned from their leaders about how to behave in the workplace, thus reducing their deviant behavior.

However, this conclusion turned out to be a little premature and has not well presented the whole logic of social learning theory (Wang et al., 2021). Indeed, according to social learning theory, the extent to which employees can reproduce leaders’ behavior is influenced by four important sub-processes, including attention, retention, motor reproduction, and motivation. More specifically, the first two sub-processes are concerned with how employees attend to, select from, and make sense of cues transmitted in leaders’ behavior, whereas the latter two focus on whether employees have enough ability and motivation to practice and maintain what they have learned from leaders (Bandura, 1972, 1977). As such, the process of social learning is more complicated than employees simply exposed to leaders and naturally learning from their behavior (Ogunfowora, 2014). Following this whole logic, this study proposes that employees do not naturally imitate ethical leaders’ behavior; instead, they need to notice and accurately make sense of ethical leaders’ behavior as well as feel fully motivated to reproduce this behavior. To be specific, we propose that LMX differentiation serves as an important boundary condition in the social learning process, since the way other members are treated by leaders is an essential factor that influences how employees observe, learn, and develop their own judgements about the extent to which they view ethical leaders as credible, attractive, and legitimate models and imitate leaders’ behavior (Bandura, 1977; Mackey et al., 2020).

### The Moderating Role of LMX Differentiation

As a group-level construct that draws on the degree of within-group variation in LMX, LMX differentiation captures the differentiated exchanges that a leader forms with different employees, ranging from higher to lower quality and differing in exchange patterns (Erdogan and Bauer, 2010; Gooty and Yammarino, 2016). More specifically, with some employees, leaders form lower-quality relationships wherein interpersonal interaction is limited to fulfilling contractual obligations, whereas, with other employees, leaders develop higher-quality relationships in which interaction patterns go beyond contractual obligations (Henderson et al., 2009). The previous research indicates that although the degree of differentiation between groups varies, LMX differentiation seems to be prevalent and usual in groups and could be noticed by employees within the group (Liden and Graen, 1980; Liden et al., 2006; Erdogan and Bauer, 2010), which will thus influence the cognitions, attitudes, and behavior of employees (Henderson et al., 2009; Martin et al., 2018). Following extant research that conceptualized LMX differentiation as a moderator rather than an independent variable (e.g., Mackey et al., 2020), this paper likewise intends to examine the moderating role of LMX differentiation. Specifically, drawing on social learning theory, we propose that high level of LMX differentiation will impede employees’ attention and interpretation of ethical leaders’ behavior and weaken their motivation to learn from leaders, therefore weakening the relationship between ethical leadership and employees’ deviant behavior.

First, compared with lower LMX differentiation, high LMX differentiation within teams implies that the leader is non-neutral, which violates the perception of equality and consistency employees hold regarding leaders’ behavior (Hooper and Martin, 2008; Harris et al., 2014). This inconsistency underlying leaders’ behavior would bring employees sense of uncertainty and loss of control in terms of their interactions and relationships with leaders, which may occupy employees with multiple negative effects (e.g., anxiety, fear, anger; Weiss et al., 1999; Barclay and Kiefer, 2019). For example, in-group employees may be afraid of losing the preference from leaders and being expelled to the “out-group,” while out-group employees may feel resentful about the unfair treatment they received. As such, immersed in negative effect, employees do not only lack enough attentional resources to observe ethical leaders’ behavior, but also tend to view and make sense of leaders’ behavior from a more negative perspective (Holtom et al., 2012) and thus are less likely to model after ethical leaders. Therefore, we propose that the refraining effect of ethical leadership on employee deviant behavior will be weakened in teams with a high level of LMX differentiation.

Second, the previous research suggests that employees are not only concerned with whether they are treated fairly themselves, but also pay attention to the overall fairness within the group (Lind, 2001). Signifying that the leaders treat employees differently, higher LMX differentiation will undermine employees’ overall perception of justice within the group and thus lead employees to suspect the trustworthiness of leaders and the true intent behind ethical leadership. This further spurs employees to hold a negative attitude toward leaders and leaders’ actions, making them less likely to view ethical leaders as legitimate and credible role models (Otken and Cenkci, 2012). As such, despite that the leaders behave ethically and fairly to some extent, employees are likely to discount this behavior due to leaders’ differentiated and unfair interactions with the whole group, and therefore less likely to model after ethical leaders, finally reducing their deviant behavior less due to ethical leadership.

Third, within teams high in LMX differentiation, employees tend to constantly notice and compare differences in LMX status among all members, spurring the process of social categorization, and thus are less likely to establish their identities as team members (Harris et al., 2014; Lai et al., 2018). Under such circumstances, employees are less inclined to see the linkage between them themselves and their teams, and therefore, less motivated to imitate leaders’ ethical behavior and put effort to refrain their harmful behavior out of interest of their teams or other members. In support of our argument, previous studies have shown that LMX differentiation will decrease employees’ awareness of leaders’ caring nature (Haynie et al., 2019), reduce the extent to which employees identify as team members, and weaken the positive effect of leaders’ behavior on employees (Harris et al., 2014). Thus, we suggest a moderating role of LMX differentiation on the relationship between ethical leadership and employee deviant behavior.

*Hypothesis 1*: LMX differentiation moderates the relationship between ethical leadership and employee deviant behavior, such that the relationship between ethical leadership and employee deviant behavior is stronger in teams with lower LMX differentiation than higher LMX differentiation.

### The Mediating Role of Psychological Empowerment

Defined as a multifaceted concept that reflects employees’ psychological and motivational states, psychological empowerment manifests through a set of four cognitions, namely meaning, self-determination, competence, and impact (Spreitzer, 1995). Specifically, meaning captures employees’ perceived value of their work goals or purpose in relation to their own ideals or standards (Thomas and Velthouse, 1990). Self-determination reflects a sense of autonomy and control in initiating and regulating work behavior and processes (Deci et al., 1989). Competence, or self-efficacy, refers to employees’ beliefs regarding their capabilities to perform their work skillfully (Bandura, 1978). Impact describes the extent to which employees feel that they can make a difference in strategic, administrative, or operational outcomes at work (Ashforth, 1989). As such, psychological empowerment is conceptualized as a higher-order latent variable that reflects through the above four dimensions (Spreitzer, 1995). In line with this conceptualization and previous studies on psychological empowerment (e.g., Seibert et al., 2004; Dust et al., 2018), this study tends to explore the role of psychological empowerment as an integrative motivational construct rather than examine the possibly unique effect of its sub-dimensions. Furthermore, according to Thomas and Velthouse (1990), employee psychological empowerment is not an enduring personality trait but shaped and influenced by the work contexts, such as leaders and leaders’ behavior. In this study, we propose that employee psychological empowerment serves as a critical mechanism wherein ethical leadership and LMX differentiation interact to affect employee psychological empowerment, such that ethical leadership contributes to enhancing employee psychological empowerment in teams with low LMX differentiation, and then, increased psychological empowerment conduces to decreasing employee deviant behavior.

According to social learning theory, individuals tend to learn through observing the behavior, values, and attitudes of those who are deemed as attractive, credible, and legitimate models (Bandura, 1972, 1977). Furthermore, social learning theory suggests that the social learning process is not purely behavioral, but also a cognitive and motivational process during which employees attend to, select from, and make sense of cues behind leaders’ behavior, and feel motivated to behave as leaders (Bandura, 1977, 1986). As hypothesized above, within teams with low LMX differentiation, employees are inclined to view ethical leaders as role models and learn from their leaders about how to perceive, understand, and approach their work, which will then influence employees’ psychological empowerment. More specifically, first, ethical leaders emphasize the moral rightness of decisions, value “the means” as opposed to “the ends” when defining success, and discuss with employees about the importance of doing the right thing rather than simply doing the practical and profitable thing (Brown et al., 2005; Brown and Trevino, 2006). In teams with lower LMX differentiation, employees are more likely to model after their leaders, and then tend to make decisions and approach tasks at work from a broader and longer-run perspective rather than simply focusing on the bottom line, and thus are more likely to experience the true meaning of their work (Dust et al., 2018). Besides, by stressing the importance of doing the right thing with the right approach, ethical leaders encourage employees to pay attention to the value and process rather than the results, and to be responsible for what they do (Brown et al., 2005; Dust et al., 2018). When LMX differentiation is low, employees are more likely to feel encouraged by leaders to take control of their decisions and behavior themselves and thus feel more sense of self-determination and competence (Walumbwa et al., 2011; Tu and Lu, 2016; Dust et al., 2018). As such, employees can sense more psychological empowerment at work by observing and imitating ethical leaders’ behavior when LMX differentiation is low.

Second, through ongoing dialogue and communication with employees about the business ethics and values, ethical leaders do not only link employees’ work with organizational goals, but also clarify how employees contribute to the achievement of socially responsible goals (Brown et al., 2005). Thus, under the condition of low LMX differentiation, employees tend to learn from their leaders that their work is meaningful to both their organizations and the whole society (Piccolo et al., 2010; Wang and Xu, 2019). Furthermore, such linkage between employees’ work and the broader context also contributes to enhancing employees’ sense of impact and competence, as it makes employees believe that they can make a positive difference to others (Zhu et al., 2004; Piccolo et al., 2010). As such, employees will feel more psychologically empowered at work.

Third, ethical leaders give employees opportunities to express their ideas, listen to what they say, and offer them more influence and discretion over decision making (Brown et al., 2005). When LMX differentiation is low, employees tend to trust in leaders and interpret leaders’ delegation as out of sincere intent to get employees involved rather than buck-passing and thus feel more psychologically empowered (Dong et al., 2020). On the one hand, through higher involvement in decision making, employees can learn about how to take control of their work, thus feeling more competence and self-determination in themselves (Zhu et al., 2004). On the other hand, deeper involvement also helps employees to regard themselves as an integral part of their organization and become aware of their indispensable roles in organizational functioning and therefore garner an increased sense of impact (Dust et al., 2018). Therefore, in line with and extending previous studies (Zhu et al., 2004; Dust et al., 2018), we draw upon social learning theory and posit that ethical leadership is more likely to enhance employees’ psychological empowerment in teams with lower LMX differentiation.

*Hypothesis 2*: Ethical leadership and LMX differentiation interact to affect employee psychological empowerment, such that ethical leadership is more likely to enhance employee psychological empowerment in teams with low LMX differentiation than in teams with high LMX differentiation.

Furthermore, in line with research on psychological empowerment, we propose that psychologically empowered employees are less likely to conduct deviant behavior. First, employees with higher psychological empowerment gain more sense of meaning from what they do and feel more confident to accomplish what they do. As a result, they experience more satisfaction attached to their job; further, they are more motivated and energized to complete their work (Carless, 2003; Seibert et al., 2011). As such, employees tend to be more absorbed in their work and are less likely to be distracted from their work to engage in deviant behavior (Bhatnagar, 2012; Shantz et al., 2016).

Second, higher psychological empowerment indicates that employees are granted more discretion in their work. They are also more involved in the team and have certain influence on the decision making, which promotes them to foster a sense of identification and commitment to their team (Liden et al., 2000; Avolio et al., 2004). As such, on the one hand, employees are more likely to enjoy working here and thus are inclined to conduct more beneficial and less detrimental behavior to their team to reciprocate for their enjoyable experiences in their team (Maynard et al., 2013). On the other hand, with a desire to remain here, they would also try to refrain themselves from committing deviant behavior for fear of being dismissed from the team (Harris et al., 2009; Seibert et al., 2011).

Third, higher psychologically empowered employees can handle the stress and adversity better and thus feel less strain and experience more positive effect, which decreases their tendencies to conduct deviant behavior (Seibert et al., 2011; Shin et al., 2012; Koopmann et al., 2019). In addition, employees who earn more psychological empowerment are also more resilient when facing challenges and adversity, and committed to longer-term goals (Luthans et al., 2010), and therefore are less likely to engage in deviant behavior since these harmful behavior are usually thought to be more short-term orientated and useless for goal achievement (Penney et al., 2011). Thus, we propose that employee psychological empowerment is conducive to decreasing their deviant behavior.

*Hypothesis 3*: Employee psychological empowerment is negatively related to their deviant behavior.

As argued above, ethical leadership is more likely to enhance employees’ psychological empowerment in teams with lower LMX differentiation and enhance employees’ psychological empowerment and then contributes to less deviant behavior. Thus, we propose that employee psychological empowerment is a critical psychological and motivational mechanism linking the interaction of ethical leadership and LMX differentiation with employees’ deviant behavior, demonstrating a pattern of mediated moderation effect between the focal variables. Hence, the following hypothesis is stated:

*Hypothesis 4*: Employee psychological empowerment mediates the interaction effect of ethical leadership and LMX differentiation on employee deviant behavior, such that the indirect effect will be stronger in teams with low LMX differentiation than in teams with high LMX differentiation.

## Materials and Methods

### Participants and Procedures

A total of 425 subordinates and 43 supervisors at two different organizations were contacted by the author team and invited to participate in our research. One of the organizations is a private estate company in southwestern China, where 138 full-time employees, as well as their direct supervisors, participated in our research project; the other one is a high school affiliated to the firm above, where 287 teachers and their direct leaders agreed to involve in this study. With strong support from top managers and human resources departments of the organizations, we were provided the name lists of participants and timetables before administering the questionnaires. At the time of conducting the survey, members of our research team explained our research purpose and promised to keep all responses confidential. Then, paper questionnaires were distributed to these participants directly by our research team and taken back immediately after participants finished them independently. All employees were asked to report their background information, perceived ethical leadership, LMX with their direct leaders, as well as their psychological empowerment at work. To reduce common method variance (Podsakoff et al., 2012), team leaders were asked to evaluate the deviant behavior of their employees. All leader–employee paired data were matched based on participants’ IDs.

Our final sample consisted of 379 subordinates and 37 supervisors (each lead a team), yielding a response rate of 89.2 and 86.0%, respectively. This high response rate was facilitated through constant communication with senior management, and the company’s willingness to give employees time during the workday to complete the surveys. Among the 37 teams, 18 are from the company and 19 are from the school. The average group size of the company sample is 3.17 (*SD*=1.54), and the range of group size is from 2 to 6. The average group size of the school sample is 18.95 (*SD*=13.13), and the range of group size is from 6 to 50. The average group size of the full sample is 10.50 (*SD*=12.13). The subordinate sample consists of 32% female and 68% male participants. Their age mainly ranges from 26 to 30 and 36 to 40, 20.3 and 18.9%, respectively. The majority (92.8%) completed junior college. The average tenure of subordinate in the current job was 4.75years (*SD*=3.59). The supervisor sample consists of 46% female and 54% male participants. Their age mainly ranges from 36 to 40 and 46 to 50, 17.5 and 17.5%, respectively. The majority (92.8%) completed junior college. The average tenure of supervisor in the current job was 7.60years (*SD*=3.46).

### Measurement

All measures were initially compiled in English. Once the list of measures was complete, the items were translated into Mandarin by a bilingual research assistant and then translated back into English by a separate bilingual research assistant (Brislin, 1970). Discrepancies were addressed through conversation within the author team. All variables were measured by the seven-point Likert scale ranging from 1=strongly disagree to 7=strongly agree.

### Ethical Leadership

Subordinates rated ethical leadership using Brown et al. (2005)‘s 10-item measure. Following past research, we conceptualized ethical leadership as a unitary, single-factor construct (Brown et al., 2005). Sample items include “my supervisor will discuss with employees about business ethics or values” and “my supervisor sets an example of how to do things the right way in terms of ethics” (*α*=0.91).

### LMX Differentiation

For LMX differentiation, the seven-item scale developed by Graen and Uhl-Bien (1995) was utilized. Employees rated their perceived LMX with their direct leader. Samples are “How well does your leader understand your job problems and needs? (from 1=not a bit to 7=a great deal)” and “How would you characterize your working relationship with your leader? (from 1=extremely ineffective to 7=extremely effective)” (*α*=0.90). Consistent with Chan (1998)‘s dispersion model and prior LMX differentiation measures (e.g., Liden et al., 2006; Henderson et al., 2009; Erdogan and Bauer, 2010), we used the variance in the individual-level LMX scores for each group to capture group-level differentiation.

### Psychological Empowerment

Psychological empowerment was measured with the 12-item scale developed by Spreitzer (1995) in this survey. Subordinates were asked to rate the level of psychological empowerment in their organizations. Example items are “The work I do is meaningful to me (Meaning),” “I am confident about my ability to do my job (Competence),” “I have significant autonomy in determining how I do my job (Self-Determination),” and “My impact on what happens in my department is large (Impact)” (*α*=0.83). Following Spreitzer (1995, 1996) and Seibert et al. (2004), we averaged scores from the four dimensions of psychological empowerment as the final score of each employee.

### Deviant Behavior

Deviant behavior was measured *via* nine items from Robinson and O'Leary-Kelly (1998). In this study, each team leader was asked to rate their subordinates’ deviant behavior independently. The deviant behavior measured in this study is mainly targeting the organization. Sample items include “this employee damages property belonging to the organizations” and “said or did something to purposely hurt someone at work” (*α*=0.92).

### Control Variables

We also included individual demographic characteristics in the analysis because these variables may affect the relationships of interest (e.g., Debus et al., 2012). We added employee’s age, gender, and education as control variables. In addition, since data were drawn from two essentially distinctive sources (i.e., firm and school), we transformed sample type into dummy variables (school=1, firm=0) and treated them as control variables in the model. At the group level, we controlled for the group-mean LMX as research has shown that average levels of LMX within a group affect employee work outcomes (Hooper and Martin, 2008). We also controlled the age, gender, and work tenure of leaders to reduce the influence of different leaders on the evaluation of outcome variables.

### Analytic Strategy

Data analysis consisted of two parts. We firstly conducted the preliminary analyses, including convergent validity testing, discriminate validity testing, intra-class correlation (ICC1) testing, and correlation analysis. Following that, we then conducted hypothesis testing.

Given the hierarchical structure of our data (i.e., subordinates nested within teams), we utilized a multi-level path analysis for our data analysis (Zhang et al., 2009; Preacher et al., 2016). The data were separated across two levels: the individual level (Level 1) and the team level (Level 2). As suggested by our theoretical model, the variables at the individual level (Level 1) were ethical leadership, psychological empowerment, and employee deviant behavior, while the variable at the team level (Level 2) was LMX differentiation. Analyses were explicitly conducted with the Mplus 7.4 (Muthén and Muthén, 2015) using maximum likelihood estimation with robust standard errors. We centered LMX differentiation at its grand mean and ethical leadership at its group mean to test the interaction effect. In order to obtain a meaningful cross-level interaction effect, we estimated the random effect of the deviant behaviors on ethical leadership, as well as the psychological empowerment on ethical leadership. Simple-slopes analysis (Aiken and West, 1991) was used to probe the interaction effect. Moderated mediation hypotheses were tested *via* Monte Carlo simulation procedures using the Rmediation add-on package for the R statistical software environment (Selig and Preacher, 2008; Tofighi and MacKinnon, 2011, 2016).

## Results

### Preliminary Analyses

Our research model consists of four variables, including LMX, ethical leadership, psychological empowerment, and deviant behavior, which are theoretically independent constructs. First, we calculated AVE and CR of all variables to ensure that our research measurement has convergent validity. Factor loadings for all items were significant, and all AVEs are above 0.5 and all CRs are above 0.7 (Nunnally, 1978; Segars, 1997), demonstrating desirable convergent validity.

Second, to ensure that our research scales have discriminate validity, we completed a multi-level confirmatory factor analysis (CFAs). Four variables used in this research constitute the four-factor baseline model. We employed a parceling technique (Bagozzi and Edwards, 1998; Little et al., 2013), creating three parcels for LMX, ethical leadership, and deviant behavior, respectively, and four parcels for psychological empowerment. As the previous research suggested, when evaluate the model fit, CFI and TLI should be greater than 0.90, and the RMSEA and SRMR should be less than 0.08 (Hu and Bentler, 1999). The fit indices revealed that the proposed four-factor model fits the data well: *χ*^2^=140.75, *df*=59, *p*<0.001, CFI=0.96, TLI=0.95, SRMR_(within)_=0.06, RMSEA=0.05. Moreover, the baseline model yielded the best results when compared against a two-factor model in which LMX, psychological empowerment, and deviant behavior were set to one latent variable, *χ*^2^=833.40, *df*=64, *p*<0.001, CFI=0.64, TLI=0.56, SRMR_(within)_=0.17, RMSEA=0.19; ∆*χ*^2^=227.81, ∆*df*=5, *p*<0.001, and a one-factor model, in which all variables were set to one latent variable, *χ*^2^=1012.11, *df*=65, *p*<0.001, CFI=0.55, TLI=0.46, SRMR_(within)_=0.17, RMSEA=0.21; ∆*χ*^2^=350.06, ∆*df*=6, *p*<0.001.

Finally, the ICC(1) of psychological empowerment is 0.13, *p*<0.001, and the ICC(1) of deviant behaviors is 0.67, *p*<0.001, which indicates that these two variables have significant nesting structure (Bliese, 2000). Therefore, this study used multi-level path model to analyze data, in order to exclude the variation of team level. The correlations and descriptive statistics for the variables in the study are shown in Table 1. At the level 1, ethical leadership is positively related to psychological empowerment (*r*=0.36, *p*<0.01), and psychological empowerment is marginally positively related to deviant behaviors (*r*=0.11, *p*<0.1). These results provided preliminary support for some of the hypothesized relationships. Complete hypothesis analysis results are reported in Tables 2–4.

**Table 1:** Means, SDs, reliability coefficients, and correlations of study variables.

Variables	*M*	*SD*	1	2	3	4	5	6	7
**Level 1**									
*Employee age*	3.65								
*Employee gender*	–	–	−0.04						
*Employee education*	2.54	0.86	−0.43^**^	−0.01					
*Sample type*	−	–	−0.13^*^	0.06	0.43^**^				
Ethical leadership	5.47	1.02	−0.08	0.04	0.04	0.13^*^	(0.91)		
Psychological empowerment	5.20	0.07	0.12^*^	0.01	0.06	0.12^*^	0.36^**^	(0.83)	
Deviant behavior	1.64	0.55	0.07	−0.09	0.02	−0.16^**^	−0.07	0.11^+^	(0.92)
**Level 2**									
*Group size*	10.50	12.13							
*Leader age*	5.57	2.15	0.03						
*Leader gender*	–	–	0.07	−0.08					
*Leader tenure*	7.60	3.46	−0.11	0.17	−0.13				
*Group-mean LMX*	5.15	0.59	0.00	0.13	−0.01	0.33			
LMX differentiation	0.85	0.34	0.29	0.09	−0.56^**^	0.26	0.07		

*N=379 subordinates representing 37 supervisory units. Employee age and leader age were categorically coded as 1=age under 26years, 2=26–30, 3=31–35, 4=36–40, 5=41–45, 6=46–50, 7=51–55, 8=56–60, and 9=over 60. Employee gender and leader gender were coded as 1=male, 0=female, and 32% employee is female; 46% leader is female. Employee education was coded as 1=high school and below, 2=junior college, 3=bachelor degree, and 4=master degree and above. Sample type was coded as 1=teacher, 0=employee, and 22% are employee. Leader tenure was measured in year. Mean values of Cronbach’s alpha coefficients were presented in parentheses along the diagonal. Italics are control variables*.

+ *p<0.1*,

* *p<0.05*,

** *p<0.01*.

**Table 2:** Results of path analysis for Model 1 (multi-level moderation model).

Predictors	Deviant behavior	
	Estimate	*SE*
Intercepts	−0.465	0.960
**Level 1**		
*Employee age*	0.011	0.013
*Employee gender*	0.000	0.049
*Employee education*	0.004	0.041
*Sample type*	−0.019	0.099
Ethical leadership	−0.009	0.031
Residual variance	0.003	0.003
**Level 2**		
*Group size*	0.428	0.198
*Group-mean LMX*	−0.004^*^	0.008
*Leader age*	−0.014	0.059
*Leader gender*	−0.229	0.216
*Leader tenure*	0.042	0.029
LMX differentiation	0.260	0.325
Residual variance	0.136^**^	0.045
**Cross-level**		
Ethical leadership×LMX differentiation	0.332^**^	0.110
Residual variance for slope^a^	0.003	0.003

*N=379 subordinates representing 37 supervisory units. Intercepts were allowed to vary across supervisory units. Italics are control variables*.

a *The slope was estimated as deviant behavior on ethical leadership*.

* *p<0.05*,

** *p<0.01*.

**Table 3:** Results of path analysis for Model 2 (multi-level moderated mediation model).

Predictors	Psychological empowerment		Deviant behavior	
	Estimate	*SE*	Estimate	*SE*
Intercepts	3.663^***^	0.257	1.756	0.027
**Level 1**				
*Employee age*	0.050^*^	0.023	0.008	0.014
*Employee gender*	−0.059	0.085	0.033	0.050
*Employee education*	−0.028	0.070	0.018	0.043
*Sample type*	0.083	0.129	−0.010	0.103
Ethical leadership	0.301^***^	0.049	−0.082^**^	0.024
Psychological empowerment			0.070^*^	0.035
Residual variance	0.369^***^	0.039	0.106^***^	0.010
**Level 2**				
*Group size*	−0.003	0.056	0.012	0.070
*Group-mean LMX*	0.004	0.004	−0.004	0.007
*Leader age*	−0.044	0.028	−0.028	0.052
*Leader gender*	−0.036	0.119	−0.237	0.216
*Leader tenure*	0.006	0.023	0.037	0.032
LMX differentiation	0.086	0.149		
Residual variance	0.034	0.039	0.197^**^	0.069
**Cross-level**				
Ethical leadership×LMX differentiation	−0.553^**^	0.162		
Residual variance for slope^b^	0.002	0.059		

*N=379 subordinates representing 37 supervisory units. Intercepts were allowed to vary across supervisory units. Italics are control variables*.

b The slope was estimated as psychological empowerment on ethical leadership.

* *p<0.05*,

** *p<0.01*,

*** *p<0.001*.

**Table 4:** Conditional indirect effects of ethical leadership on deviant behavior *via* psychological empowerment at low and high levels of LMX differentiation.

	Indirect effect	95% CI
Low LMX differentiation (−1*SD*)	0.034^*^	[0.001, 0.071]
High LMX differentiation (+1*SD*)	0.008	[−0.002, 0.024]
Difference	−0.026^*^	[−0.061, −0.001]

*N=379 subordinates representing 37 supervisory units. The 95% CI for the conditional effects were calculated using Monte Carlo bootstrapping with 20,000 repetitions*.

* *p<0.05*.

### Hypotheses Testing

**Figure 2:**
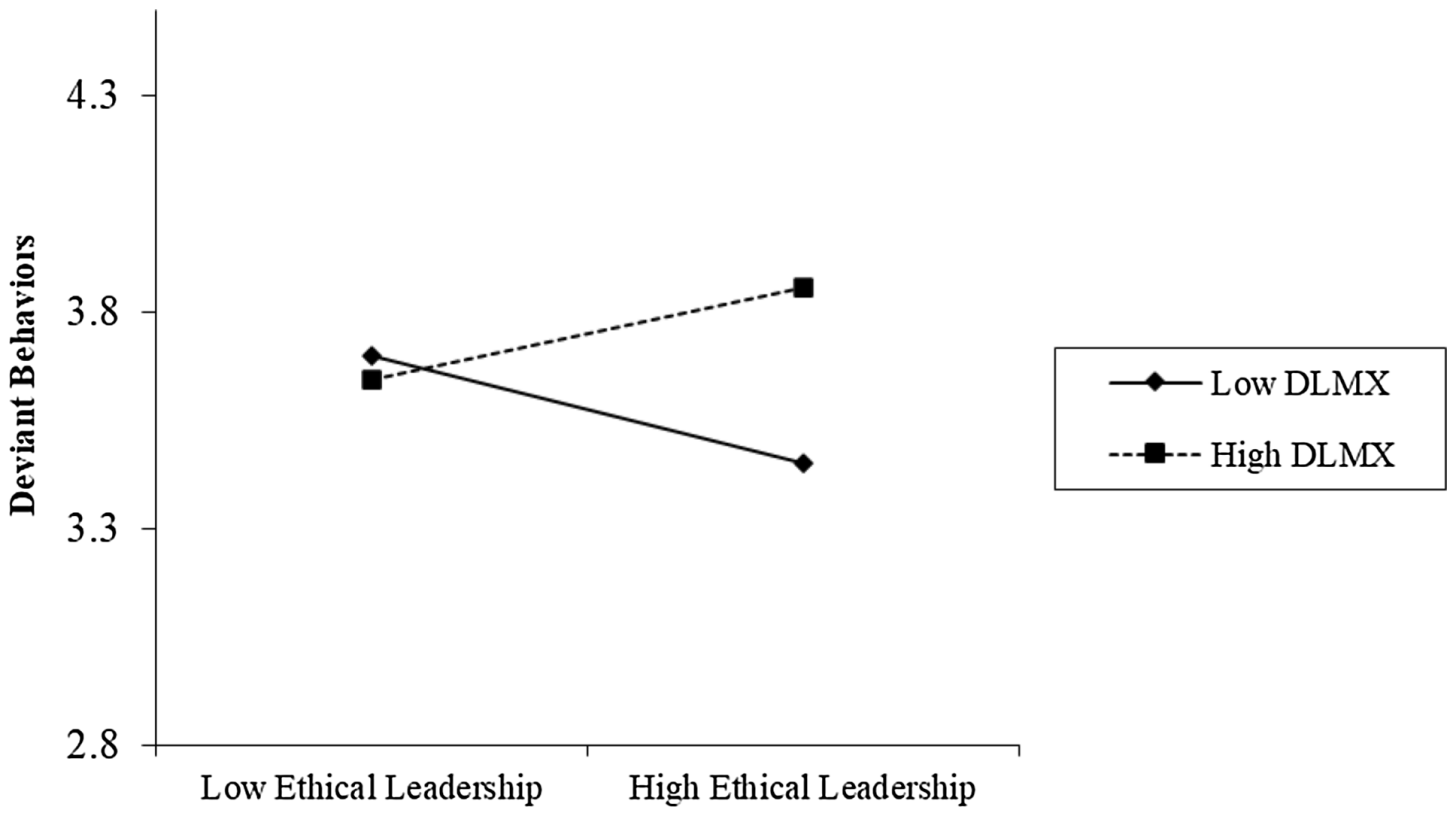
Moderating effect of LMX differentiation on the relationship between ethical leadership and deviant behaviors.

We employed path model analysis to test all hypotheses. To test Hypothesis 1, we first specified a multi-level moderation model (Model 1). Hypothesis 1 predicts that LMX differentiation moderates the relationship between ethical leadership and employee deviant behavior, such that the negative relationship between ethical leadership and employee deviant behavior is stronger in teams with lower LMX differentiation than higher LMX differentiation. Results (Table 2) showed that LMX differentiation moderated the effects of ethical leadership on deviant behavior (*γ*=0.332, *p*<0.01). Simple slope tests demonstrated that the relationship between ethical leadership and deviant behavior was negative and significant when the level of LMX differentiation was low (1 *SD* below the mean; *γ*=−0.122, *p*<0.001), but was not significant when the level of LMX differentiation was high (1 *SD* above the mean; *γ*=0.104, *p*=0.077), and the difference between low and high LMX differentiation is significant (*γ*=0.226, *p*<0.01). The interaction pattern, as shown in Figure 2, was consistent with Hypothesis 1. Thus, Hypothesis 1 was supported.

**Figure 3:**
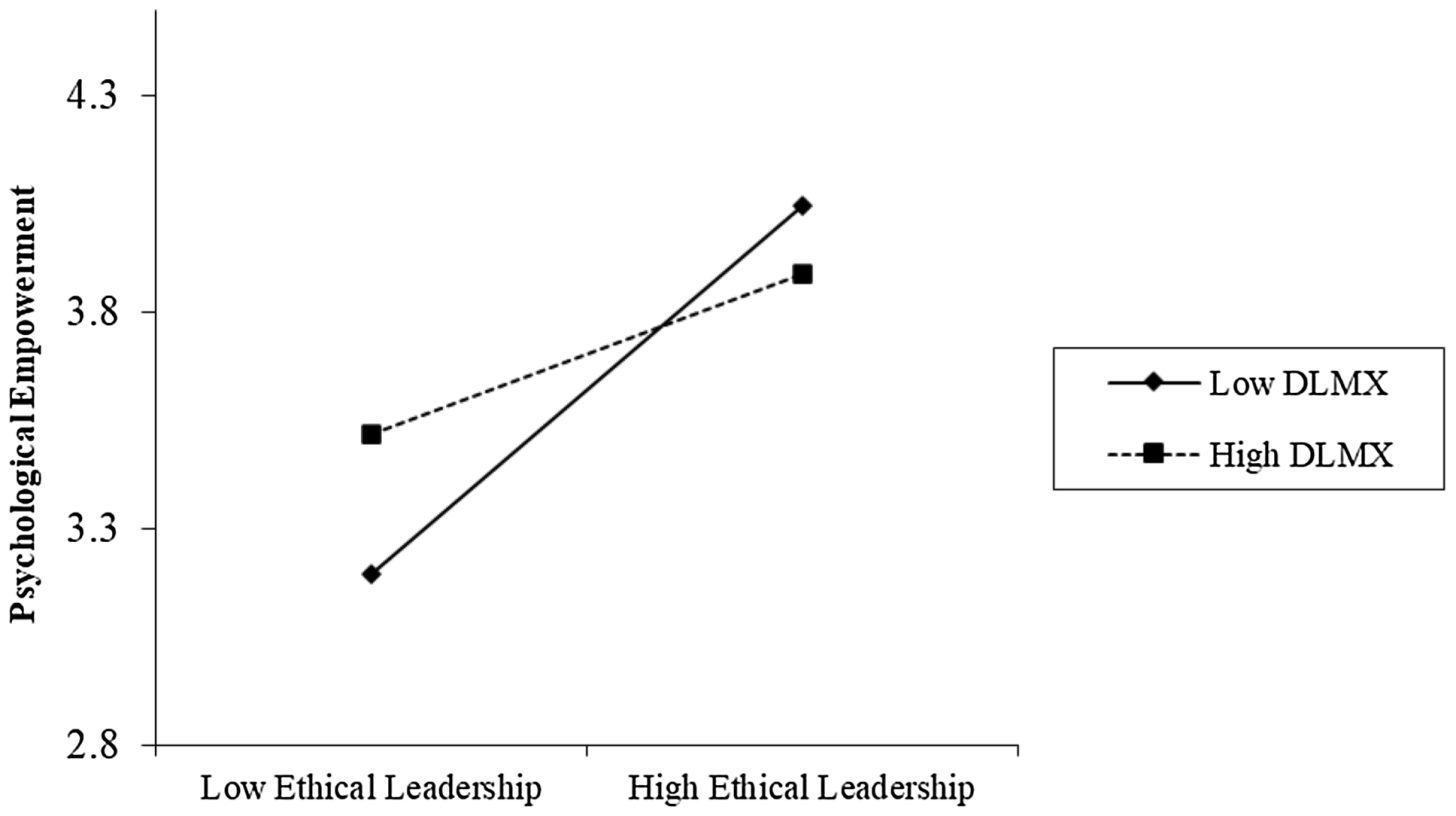
Moderating effect of LMX differentiation on the relationship between ethical leadership and psychological empowerment.

We then specified a multi-level moderated mediation model (Model 2) to test Hypothesis 2–4. Hypothesis 2 predicts that LMX differentiation moderates the relationship between ethical leadership and employee psychological empowerment, such that the negative relationship between ethical leadership and employee psychological empowerment is stronger in teams with lower LMX differentiation than higher LMX differentiation. Results (Table 3) showed that LMX differentiation moderated the effects of ethical leadership on psychological empowerment (*γ*=−0.553, *p*<0.01). Simple slope tests demonstrated that the relationship between ethical leadership and psychological empowerment was significantly stronger among teams with low LMX differentiation (1 *SD* below the mean; *γ*=0.489, *p*<0.001) than with high LMX differentiation (1 *SD* above the mean; *γ*=0.113, *p*<0.05), and the difference between low and high LMX differentiation is significant (*γ*=−0.376, *p*<0.01). The interaction pattern shown in Figure 3 was consistent with Hypothesis 2. Thus, Hypothesis 2 was supported.

In addition, Hypothesis 3 is supported in Table 3 with the direct effect of psychological empowerment on deviant behavior being 0.07 (*p*<0.05). Hypothesis 4 proposed that psychological empowerment would mediate the interactive effect of ethical leadership and LMX differentiation on deviant behaviors. To explore the mediated moderation effect, we multiplied the coefficients for simple slopes generated in H3 by the coefficients for the path between psychological empowerment and deviant behavior to obtain estimates for the indirect effects. With 20,000 Monte Carlo replications, results (Table 4) showed that the indirect effects of ethical leadership on deviant behaviors were significant when the level of LMX differentiation was low (indirect effect=0.034; 95% CI=[0.001, 0.071]), but was not significant when the level of LMX differentiation was high (indirect effect=0.008; 95% CI=[−0.002, 0.024]). Moreover, the difference between these two conditional indirect effects was significant (indirect effect=−0.026; 95% CI=[−0.061, −0.001]), thereby demonstrating support for Hypothesis 4.

## Discussion

Drawing upon social learning theory, this study examined the effect of ethical leadership on employee deviant behavior. First, in accordance with our conceptual analysis, we found that ethical leadership contributed to less employee deviant behavior in teams with lower LMX differentiation than higher LMX differentiation. Second, ethical leadership is related to higher psychological empowerment in teams with lower LMX differentiation, and increased psychological empowerment is related to less deviant behavior. Finally, we also found a mediated moderation effect; namely, the interactive effect of ethical leadership and LMX differentiation on employee deviant behavior is mediated by employee psychological empowerment. These findings generate several theoretical and managerial implications.

### Theoretical Implications

Our findings contribute to research on ethical leadership, psychological empowerment, and deviant behavior in at least three ways. First, we examine the boundary effect of LMX differentiation on the relationship between ethical leadership and employee deviant behavior and found that ethical leadership is more likely to decrease employee deviant behavior in teams with lower LMX differentiation. The previous research has generally suggested that ethical leadership is related to less deviant behavior (e.g., Mayer et al., 2009; Resick et al., 2013); however, recent studies found that this relationship does not always hold (e.g., Wang et al., 2021). In response to existing inconsistent findings, our study suggests that the social learning process wherein employees model after ethical leaders do not happen necessarily, but rather, is influenced by the broader context wherein leaders interact with other members. As such, we challenge the generally held assumption that employees necessarily learn from ethical leaders and find a possible explanation for recent inconsistent findings in terms of the relationship between ethical leadership and deviant behavior. This helps us to probe when ethical leadership is more or less effective in decreasing workplace deviance and thus better understand the complicated relationship between ethical leadership and deviant behavior.

Second, our study illustrates the critical mediating role of employee psychological empowerment in the interactive effect of ethical leadership and LMX differentiation on employee deviant behavior. Although the relationship between ethical leadership and psychological empowerment, as well as the relationship between psychological empowerment and deviant behavior, is nearly well established in extant research (e.g., Kim et al., 2016; Dust et al., 2018), how psychological empowerment takes effect in the relationship between ethical leadership and deviant behavior is still understudied. Thus, in order to probe how the social learning process unfolds under the condition of low LMX differentiation, we explore the mediating effect of psychological empowerment. This helps us to better and more comprehensively understand the underlying psychological and motivational states of employees when ethical leadership and LMX differentiation jointly affect employee deviant behavior.

Third, drawing upon social learning theory as the overarching framework, we investigate how and under what conditions employees are more likely to take ethical leaders as role models and learn from them. Social learning theory suggests that the social learning process is not just a behavioral process, but also a cognitive and motivational process wherein employees attend to, select from, and make sense of cues behind leaders’ behavior, and obtain the motivation to take actions to learn from leaders (Bandura, 1977, 1986). Our study found that during this process, employees tend to extract information from how other members are treated by leaders, based on which to interpret ethical leaders’ behavior and acquire sense of psychological empowerment to refrain from deviant behavior. As such, we apply social learning theory to understand the effect of ethical leadership from a more comprehensive and nuanced perspective and, meanwhile, make an extension to social learning theory by identifying the important boundary effect of LMX differentiation during the social learning process.

### Practical Implications

The findings of this study also offer some useful insights to practitioners. First, this study highlights the important role of ethical leadership in reducing employee deviant behavior, which provides a theoretical basis for organizations to foster ethical leadership in order to reduce workplace deviance. More specifically, organizations can utilize a morality test to identify, select, and promote candidates who demonstrate the characteristics of a moral person and a moral manager for managerial positions. In addition, they can also invest in programs to encourage leaders to comply with high moral standards and develop leaders’ ethical leadership capabilities.

Second, our study indicates that the refraining effect of ethical leadership on employee deviant behavior is conditional on LMX differentiation, and a high level of LMX differentiation could attenuate the positive effect of ethical leadership. The findings alert leaders to be aware of their interactions with all employees and to make sure that every employee feels fairly and equally treated. Despite the fact that it might be unavoidable for leaders to differentiate between “in-group” and “out-group” members due to their limited time and energy, it is important for leaders to notice the costs of such differentiation and endeavor to control the corresponding negative impact. For instance, leaders could provide adequate explanations and a reasonable basis for their differentiated actions to offset the negative impact of LMX differentiation, as the previous research shows that employees seem to view task performance as a legitimate standard for leaders to depend on in developing differentiated relationships with employees (Chen et al., 2018).

Third, our finding shows that the interactive effect of ethical leadership and LMX differentiation on employee deviant behavior is achieved by enhancing their psychological empowerment. This suggests that leaders should pay attention to the psychological needs of employees, discuss with them about issues that specifically concern them, and listen to their ideas and opinions when making decisions so as to increase their feeling of psychological empowerment and, further, to decrease their deviant behavior.

### Limitations and Directions for Future Research

Despite the above contributions, some limitations of this study should be noted, which suggests meaningful directions for future research. First, this study is based on a cross-sectional research design, which could not ensure conclusions regarding causality. Although we introduce a multi-source approach when collecting data and some of the effects of our model are less likely to work the other way around (for instance, it seems impossible and of little theoretical significance to argue that employee psychological empowerment leads to their perception of ethical leadership), future research could be based on more rigorous research design, like an experiment or a longitudinal research design, to claim causality.

Second, consistent with the original conceptualization and previous studies on psychological empowerment (e.g., Seibert et al., 2004; Dust et al., 2018), we operationalize psychological empowerment as an overall construct with four dimensions of *meaning*, *competence*, *self-determination*, and *impact* and tend to explore the role of this integrative motivational construct rather than examine the possibly unique effect of its sub-dimensions. Nevertheless, in order to understand the construct of psychological empowerment deeper, future research could also differentiate these four dimensions and explore whether differences exist regarding the effect of ethical leadership on these four dimensions as well as the effect of these four dimensions on employee deviant behavior. Furthermore, recent studies have noted that there might be some possible dark sides of psychological empowerment (e.g., Luth, 2012); thus, although this is beyond what we would like to discuss in this paper, we suggest that future studies can probe its possible dark sides to promote research on psychological empowerment.

Third, in our study, we have found consistent results between two different types of organizations (i.e., company and school), providing more robust evidence for our results and initial cues about the generalizability of this result. However, future research can also survey more types of organization from different industries to examine the hypothesized relationship and to see whether there were any significant and interesting differences across industries. Furthermore, future research can also examine the hypothesized effect in different countries to see whether there are any differences. For example, the two organizations in our study are both from China wherein people conform more to the equality principle, emphasizing solidarity and harmony among team members, and thus, the violations of equality due to LMX differentiation are more detrimental. However, for those from more individualistic cultures, they are more likely to hold an equity principle, preferring distinguished treatments from leaders within the team (Yu et al., 2018). As such, the impeding effect of LMX differentiation on the relationship between ethical leadership and employee deviant behavior may be weaker.

## Data Availability Statement

The raw data supporting the conclusions of this article will be made available by the authors, without undue reservation.

## Ethics Statement

Ethical approval was not required for this study, in accordance with the local legislation and institutional requirements, because this study only required the subjects to objectively evaluate the behavior of their leader and the atmosphere of the team, and did not involve any ethical issues. The patients/participants provided their written informed consent to participate in this study.

## Author Contributions

All authors contributed to the study conceptualization and research design. YG and LC proposed the research. LS and YG collected and analyzed the data. YG and LC written the first draft of the manuscript. LS and XZ commented on previous versions of the manuscript and revised it critically for important intellectual content. All of the authors read and approved the final manuscript.

## Funding

This study was partially supported by the National Natural Science Foundation of China (Grant No. 72002113, No. 71771133, No. 71772176). The funding body facilitates the authors’ data collection, data analysis and writing.

## Conflict of Interest

The authors declare that the research was conducted in the absence of any commercial or financial relationships that could be construed as a potential conflict of interest.

## Publisher’s Note

All claims expressed in this article are solely those of the authors and do not necessarily represent those of their affiliated organizations, or those of the publisher, the editors and the reviewers. Any product that may be evaluated in this article, or claim that may be made by its manufacturer, is not guaranteed or endorsed by the publisher.
